# Potential of Tetracycline Resistance Proteins To Evolve Tigecycline Resistance

**DOI:** 10.1128/AAC.02465-15

**Published:** 2016-01-29

**Authors:** Marius Linkevicius, Linus Sandegren, Dan I. Andersson

**Affiliations:** Department of Medical Biochemistry and Microbiology, Uppsala University, Uppsala, Sweden

## Abstract

Tigecycline is a glycylcycline antibiotic active against multidrug-resistant bacterial pathogens. The objectives of our study were to examine the potential of the Tet(A), Tet(K), Tet(M), and Tet(X) tetracycline resistance proteins to acquire mutations causing tigecycline resistance and to determine how this affects resistance to earlier classes of tetracyclines. Mutations in all four *tet* genes caused a significant increase in the tigecycline MIC in Escherichia coli, and strains expressing mutant Tet(A) and Tet(X) variants reached clinically relevant MICs (2 mg/liter and 3 mg/liter, respectively). Mutations predominantly accumulated in transmembrane domains of the efflux pumps, most likely increasing the accommodation of tigecycline as a substrate. All selected Tet(M) mutants contained at least one mutation in the functionally most important loop III of domain IV. Deletion of leucine 505 of this loop led to the highest increase of the tigecycline MIC (0.5 mg/liter) among Tet(M) mutants. It also caused collateral sensitivity to earlier classes of tetracyclines. A majority of the Tet(X) mutants showed increased activity against all three classes of tetracylines. All tested Tet proteins have the potential to acquire mutations leading to increased MICs of tigecycline. As *tet* genes are widely found in pathogenic bacteria and spread easily by horizontal gene transfer, resistance development by alteration of existing Tet proteins might compromise the future medical use of tigecycline. We predict that Tet(X) might become the most problematic future Tet determinant, since its weak intrinsic tigecycline activity can be mutationally improved to reach clinically relevant levels without collateral loss in activity to other tetracyclines.

## INTRODUCTION

Tigecycline has become increasingly important in treating infections, since it is one of the few antibiotics which is still effective against rapidly emerging multidrug-resistant Gram-positive and Gram-negative pathogens (1). It belongs to the group of tetracyclines called glycylcyclines (2, 3). Tigecycline is a semisynthetic antibiotic that binds to 16S rRNA and prevents successful decoding of mRNA (4–6). A bulky side chain attached to the C-9 position of ring D is the chemical improvement, compared to earlier-class tetracyclines, that led to enhanced binding to the target and evasion of common tetracycline resistance mechanisms (2, 3).

There are three main tetracycline resistance mechanisms conferred by Tet proteins (7). First, tetracycline-specific efflux proteins that belong to the major facilitator superfamily can effectively reduce the intracellular concentration of tetracycline by exporting it into the periplasm. However, the Tet(B) transporter, for example, is not able to expel tigecycline out of the cytoplasm (2, 8) most likely due to the chemical modification at position C-9. Second, bacteria can encode ribosomal protection proteins that are able to rescue inhibited ribosomes and restore protein synthesis. Ribosomal protection proteins belong to the group of GTP-hydrolyzing enzymes that includes bacterial translation elongation factors (7, 9). Tigecycline is insensitive to the activity of ribosomal protection proteins (2), and recent cryoelectron microscopy models suggest that tigecycline is able to stay bound to 16S rRNA in the presence of Tet(M), because its C-9 side chain is overlapping with the Tet(M) binding site on the ribosome (6, 10, 11). The third mechanism of tetracycline resistance involves enzymatic inactivation of the drug. Tet(X) is the most studied tetracycline-modifying enzyme, and it is active against all classes of tetracyclines, including tigecycline (12–14). For some time, it was considered a less common resistance mechanism, but a recent report from Sierra Leone demonstrated that the Tet(X) enzyme is now present in many bacterial pathogens causing human infections (15). In addition to Tet-specific resistance mechanisms, overexpression of resistance-nodulation-division (RND) family pumps was implicated in increased tigecycline resistance in members of Enterobacteriaceae (8, 16–20).

To be able to rationally and effectively deal with antibiotic resistance, we need to have conceptual and methodological tools available for prediction of when, where, and how resistance will develop when antibiotic pressures are applied. Experimental evolution is a useful approach to examine the potential of an organism to acquire mutations and/or new genes that confer an increase in resistance (21–23). Thus, in this study, we evaluated the potential of different types of Tet determinants [Tet(A), Tet(K), Tet(M), and Tet(X)] to improve their activities against tigecycline. This choice was based on the inclusion of different types of mechanisms and *tet* genes that have not been previously studied. Thus, even though it is clinically important, Tet(B) was not included, because a previous study already examined its potential to acquire mutations that confer tigecycline resistance (24). We also determined the effects that the improved activity against tigecycline had on the earlier classes of tetracyclines (tetracycline, doxycycline, and minocycline) to identify potential activity trade-offs. In addition to identifying novel resistance mutations and trade-offs, our results also demonstrate how one can perform an experimental analysis of the risk that existing genes will acquire expanded capabilities to cause resistance.

## MATERIALS AND METHODS

### Bacteria, Tet determinants, and expression vectors.

Representative *tet* genes from efflux [*tet*(A) and *tet*(K)], ribosomal protection [*tet*(M)], and enzymatic modification [*tet*(X)] groups were chosen for this study. The pUUH239.2 plasmid (GenBank accession number NC_016966) from Klebsiella pneumoniae (25) was used as a template for the *tet*(A) gene, with the primary start codon GTG (26). Plasmid pT181 (GenBank accession number NC_006629) from Staphylococcus aureus COL was a template for the *tet*(K) gene, and chromosomal DNA of S. aureus Mu50 (GenBank accession number NC_002758) was a template for the *tet*(M) gene. Both *tet*(K) and *tet*(M) determinants were a gift from Alex O'Neill. The *tet*(X) gene sequence from Bacteroides thetaiotaomicron transposon CTnDOT (GenBank accession number AJ311171.1) was used to synthesize *tet*(X) by GenScript USA, Inc. All *tet* genes were amplified with primers (see Table S1 in the supplemental material) that added ribosomal binding sites and restriction sites for EcoRI (upstream of coding sequence) and XbaI (downstream of coding sequence). PCR products of *tet* genes were digested and cloned into the pBAD30 vector between the EcoRI and XbaI sites under expression control of the P_BAD_ promoter, which increased the expression level 100- to 1,000-fold after induction with arabinose (27). This is comparable to the expression levels reported for Tet(B) expressed from the natural promoter (28). Ligated constructs were transformed into Escherichia coli DH5α (DA17737) and selected on Luria-Bertani agar (LA) supplemented with 100 mg/liter of ampicillin.

### Mutant library generation.

In order to generate the libraries of mutagenized *tet*(A), *tet*(K), *tet*(M), and *tet*(X) sequences, 10 separate error-prone PCRs per gene were run with AmpliTaq Gold DNA polymerase (Applied Biosystems) in mutagenic buffer (29). In short, a 50-μl PCR mixture contained approximately 10 ng of linearized plasmid DNA, 1× AmpliTaq Gold PCR buffer (Applied Biosystems), 1.5 mM MgCl_2_ (Applied Biosystems), 0.2 mM deoxynucleoside triphosphates (dNTPs; Thermo Scientific), 0.3 μM forward primer and 0.3 µM reverse primer (see Table S1 in the supplemental material), and 5 U of DNA polymerase. In addition, the PCR mixture was supplemented with 4 or 6 μl of mutagenic buffer (4 mM dCTP [Thermo Scientific], 4 mM dTTP [Thermo Scientific], 27.5 mM MgCl_2_ [Thermo Scientific], and 2.5 mM MnCl_2_) to vary the mutation rate. The PCR mixture was subjected to 2 min of initial denaturation at 94°C followed by 25 cycles of 1 min of denaturation at 94°C and 2 min of annealing and elongation at 72°C. A final elongation step was performed for 10 min at 72°C. After confirmation of PCR products with agarose gel electrophoresis, 10 reaction products were pooled to form the *tet* sequence library. Two sequence libraries (using 4 or 6 μl of mutagenic buffer) were generated per Tet determinant.

### Mutant selection.

The libraries were cloned into the pBAD30 vector, as described above. High-copy-number vector pUCBAD (30) was used for *tet*(M) selection experiments due to the low resistance level to tetracycline produced from pBAD30 for this gene. The ligated constructs were electroporated into E. coli NEB5α (New England BioLabs) cells and plated on Mueller-Hinton agar (MHA) plates supplemented with either 100 mg/liter of ampicillin or 10 mg/liter of tetracycline and 0.1% [*tet*(A) and *tet*(K)], 0.2% [*tet*(M)], or 0.4% [*tet*(X)] l-arabinose for library evaluation. The same culture was also selected on MHA plates supplemented with 100 mg/liter of ampicillin, 0.1% [*tet*(A) and *tet*(K)], 0.2% [*tet*(M)], or 0.4% [*tet*(X)] l-arabinose and 2× and 4× the MIC of tigecycline of the strain carrying the respective unmutagenized *tet* construct. E. coli DH5α expressing unmutagenized *tet* constructs had the following tigecycline MICs: 0.25 mg/liter for *tet*(A), 0.064 mg/liter for *tet*(K) and *tet*(M), and 0.19 mg/liter for *tet*(X). After 24 h and 48 h of incubation at 37°C, the colonies were purified on MHA plates with the corresponding supplements, as for selection. Only plasmids of the mutants that grew in the last isolation step were prepared using the E.Z.N.A plasmid minikit I (Omega Bio-Tek) and retransformed to new E. coli DH5α cells. If retransformants demonstrated increases in the tigecycline MIC (at least 2-fold), it was concluded that the observed increase was due to mutation accumulation in the *tet* gene and not because of spontaneous chromosomal mutations. The sequences of such *tet* genes were determined by DNA sequencing, as described below.

### Reconstruction of *tet* mutations.

In order to evaluate the effect of specific mutations on the increased tigecycline MICs, inverse PCR mutagenesis (31) was used to construct mutations in the plasmids carrying *tet* genes. Briefly, the mutated nucleotides were designed to be at the end of either the forward or reverse primer sequence (see Table S1 in the supplemental material). A PCR using Phusion high-fidelity DNA polymerase (Thermo Scientific) was performed to synthesize a linear plasmid with the desired mutation present at one of the ends. The primers were phosphorylated with T4 polynucleotide kinase (Thermo Scientific) to be able to circularize the plasmid after PCR. The PCR mixture (50 μl) contained 1 μl of boiled overnight culture as a template mixed with 1× Phusion HF buffer (Thermo Scientific), 0.2 mM dNTPs (Thermo Scientific), 0.5 μM phosphorylated forward primer and 0.5 µM reverse primer, and 0.012 U of DNA polymerase. Linear plasmids with a desired mutation present were digested with DpnI (Thermo Scientific) to cut the remaining DNA template, and blunt-end ligation was performed using T4 DNA ligase (Thermo Scientific). All reconstructed mutations were verified by DNA sequencing.

### Determination of MICs.

MICs of *tet* mutants were measured using Etest strips (bioMérieux). A culture was incubated in a shaking incubator (190 rpm) at 37°C overnight and then diluted 100-fold in 0.9% NaCl (wt/vol) solution. The diluted suspension was distributed on an MHA plate supplemented with different concentrations of l-arabinose (0.1% to 0.4%). An Etest strip was applied, and the plate was incubated at 37°C for 16 to 20 h. The MIC was read as the lowest concentration at which no bacterial growth was observed.

### DNA sequencing.

Target genes were PCR amplified with screening primers binding upstream and downstream of the *tet* coding region using 2× the PCR mix (Thermo Scientific). PCR products were purified with the GeneJet gel extraction kit (Thermo Scientific) and premixed with one of the sequencing primers. The mixture was sent to Eurofins MWG Operon (Ebersberg, Germany) for sequencing. For the list of primers used, refer to Table S1 in the supplemental material.

## RESULTS

### Ability of naive nonmutated *tet* genes to confer resistance to different classes of tetracyclines.

**FIG 1:**
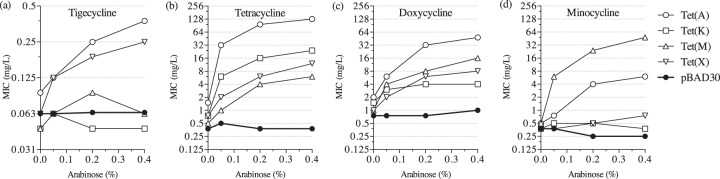
MICs of tetracyclines in E. coli expressing unmutagenized Tet proteins: tigecycline (a), tetracycline (b), doxycycline (c), and minocycline (d).

We chose four Tet determinants, representing the main tetracycline resistance mechanisms, to evaluate their ability to protect E. coli cells from different classes of tetracyclines. The MICs of tigecycline, tetracycline, doxycycline, and minocycline were measured for the strains harboring unmutagenized variants of Tet(A) and Tet(K) efflux pumps, ribosomal protection protein Tet(M), and the modifying enzyme Tet(X) (Fig. 1). These enzymes were expressed at different levels via the use of the arabinose-inducible P_BAD_ promoter, allowing us to regulate gene expression in a controllable manner. Overexpression of the efflux pump Tet(A) and modification enzyme Tet(X) provided a low-level increase in the MIC of tigecycline (0.25 to 0.38 mg/liter at the highest expression conditions), while no increase in the tigecycline MIC was observed during expression of the Tet(K) efflux pump or the Tet(M) ribosomal protection protein (Fig. 1a). As expected, all four determinants demonstrated increased MICs of tetracycline in response to arabinose induction, with efflux pumps giving the highest resistance (Fig. 1b). Similar to tetracycline, all tested determinants conferred increased resistance against doxycycline (Fig. 1c). For minocycline, Tet(M) provided the best protection, while minocycline MICs of the Tet(K) and Tet(X) strains were similar to the level of the empty vector control (Fig. 1d). Overall, Tet(A) had the best activity against the majority of tested tetracyclines, and the Tet(K) pump was the poorest resistance determinant. In addition, none of the unmutagenized Tet determinants reached the clinical breakpoints set by the European Committee on Antimicrobial Susceptibility Testing of ≤1 mg/liter (sensitive) and >2 mg/liter (resistant) (32), or by the Food and Drug Administration of ≤2 mg/liter (sensitive) and ≥8 mg/liter (resistant) (33), when expressed at a high level from a nonnative promoter.

### Potential of the Tet(A) efflux pump to develop tigecycline resistance.

**FIG 2:**
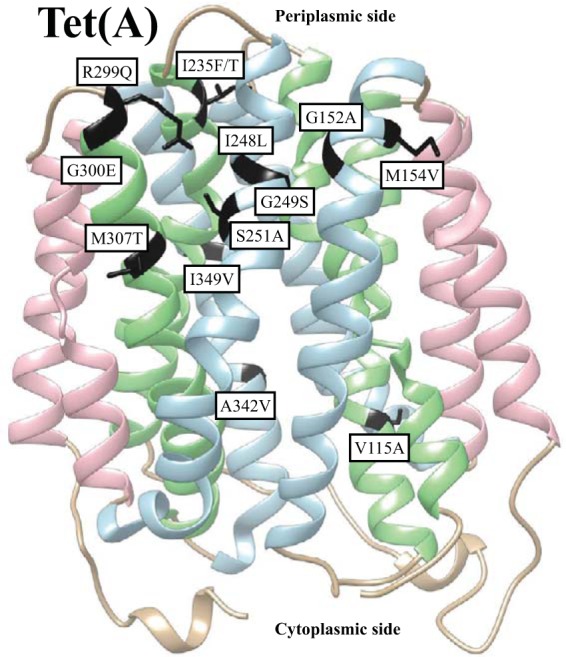
Location of tigecycline resistance mutations in Tet(A): hydrophobic putative transmembrane (TM) regions (pink), completely amphiphilic putative TM regions (light blue), and partially amphiphilic putative TM regions (light green) are indicated. Mutations mentioned in the text are marked in black. Prediction of tertiary structure was performed using online protein homology/analogy recognition engine V 2.0 (Phyre2; http://www.sbg.bio.ic.ac.uk/phyre2/html/page.cgi?id=index) with the intensive modeling mode.

We explored the capacity of the Tet proteins to accumulate mutations leading to even higher MICs of tigecycline. During the selection experiments, two libraries (TETA4L2 and TETA6L1) of mutated *tet*(A) sequences were screened, generating approximately 8 × 10^6^ transformants per library (see Table S2 in the supplemental material). To evaluate the mutation frequency, we compared the transformation efficiency on tetracycline plates [measuring functional *tet*(A) genes after mutagenesis] with the transformation efficiency on ampicillin (measuring total transformants). On average, 7 of 10 transformants carried a Tet(A) pump that retained tetracycline resistance after mutagenesis. In total, we selected 53 independent mutants with elevated tigecycline MICs (1 to 1.5 mg/liter) during the Tet(A) selections, with 33 of them being unique (Fig. 2; see also Table S3 in the supplemental material). Control experiments showed that the increased MICs were the result of mutations in the plasmid-borne *tet* genes. The most common amino acid substitution identified in 17 unique mutants was G300E, located in the putative periplasmic loop P5. Other frequent changes identified in the independent mutants more than once were S251A, M154V, and I235F. They were located in the upper parts of putative transmembrane α helices TM8, TM5, and TM7, respectively. The vast majority of amino acid substitutions were located in the transmembrane regions TM2, TM4, TM5, TM7, TM8, TM10, and TM11. Transmembrane regions TM5, TM7, TM8, and TM11 contained clusters of several amino acid changes.

**FIG 3:**
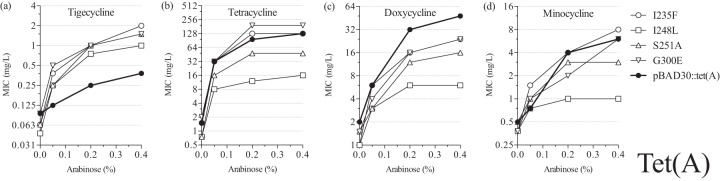
MICs of tetracyclines in E. coli expressing mutant Tet(A) proteins: tigecycline (a), tetracycline (b), doxycycline (c), and minocycline (d).

Mutations that were independently observed in the selection experiments more than once were chosen for further characterization. We measured the MICs of the four different tetracyclines for the subset of Tet(A) mutants (Fig. 3), and all of them showed increased MICs of tigecycline compared to the unmutagenized control, reaching 1 to 2 mg/liter at the highest *tet*(A) expression conditions (Fig. 3a). As tigecycline has different properties due to its distinct chemical structure, it was not surprising that some mutations that caused an increase in the MIC of tigecycline were associated with a functional trade-off for the other drugs. Thus, lower MICs of tetracycline and minocycline were observed in S251A and I248L mutants, while the MICs of doxycycline dropped in all mutants tested compared to the unmutagenized control. Together, these results suggest that achieving a significant increase in tigecycline resistance is possible by accumulating mutations in the Tet(A) pump, but these changes in protein structure may also lead to a drop in the efficiency of transporting earlier classes of tetracycline antibiotics.

### Potential of the Tet(K) efflux pump to develop tigecycline resistance.

Next, we screened libraries (TETK4L2 and TETK6L2) of mutagenized Tet(K) sequences for increased tigecycline resistance (see Table S2 in the supplemental material). Unlike the Tet(A) pump, overexpression of the unmutagenized Tet(K) efflux protein did not cause any increase in the tigecycline MIC (Fig. 1a). Of 2.7 × 10^6^ transformants screened during our selection experiments, we found 3 mutants (with up to 3 individual amino acid changes) with a small but significant increase in tigecycline MIC (see Table S3 in the supplemental material). Like the Tet(A) selections, these amino acid substitutions accumulated in transmembrane segments of the pump. Mutation Y58H was isolated in two independent mutants, and it was located in TM2. The rest of the identified mutations were found in the putative transmembrane regions TM1, TM2, TM7, cytoplasmic loop C1, and the marginal area of TM4 and C2.

**FIG 4:**
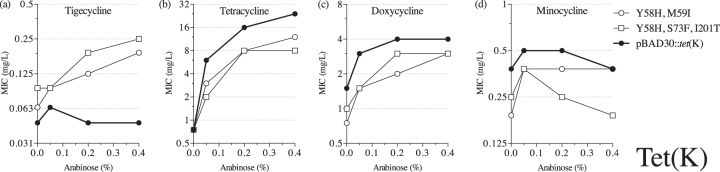
MICs of tetracyclines in E. coli expressing mutant Tet(K) proteins: tigecycline (a), tetracycline (b), doxycycline (c), and minocycline (d).

We chose two selected mutants carrying the reoccurring Y58H amino acid substitution for further analysis. While these mutants demonstrated modestly elevated tigecycline MICs [0.19 to 0.25 mg/liter at the highest *tet*(K) expression conditions] (Fig. 4a), the resistance to other tetracyclines was reduced below the unmutagenized control level (Fig. 4b to d), indicating that tigecycline-resistant Tet(K) mutants are more difficult to select than Tet(A) mutants and that increased tigecycline activity reduces Tet(K) activity against other tetracyclines.

### Potential of the Tet(M) ribosomal protection protein to develop tigecycline resistance.

**FIG 5:**
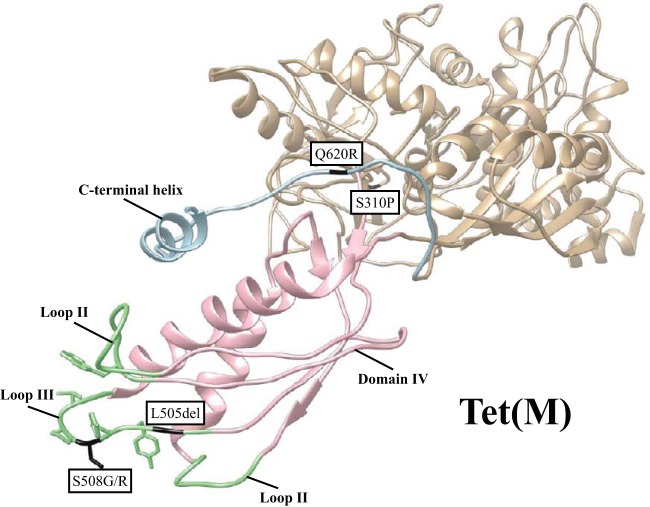
Location of mutations causing reduced susceptibility to tigecycline in Tet(M): domain IV (pink), loops I to III (light green), and C-terminal extension (light blue) are indicated. Mutations mentioned in the text are marked in black. The cryoelectron microscopy structure of Tet(M) (PDB accession number 3J9Y) is based on data from reference 11.

Ribosomal protection is the second most common clinical tetracycline resistance mechanism. Therefore, we investigated whether mutant variants of Tet(M), one of the better characterized ribosomal protection proteins, could confer resistance to tigecycline. In total, we screened 2 × 10^7^ transformants harboring mutagenized *tet*(M) sequences from two libraries (TETM4L2 and TETM6L2). During our screen, we identified 13 unique Tet(M) mutants with increased tigecycline MICs (Fig. 5; see also Table S3 in the supplemental material). All of the selected mutants carried more than one amino acid substitution; substitutions were located in domains I to V and the C-terminal extension (CTE).

**FIG 6:**
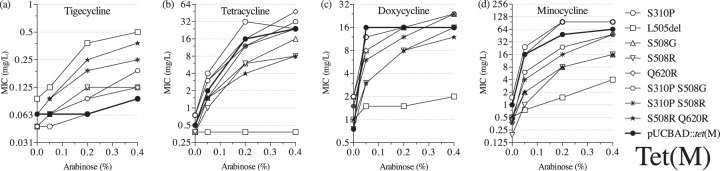
MICs of tetracyclines in E. coli expressing mutant Tet(M) proteins: tigecycline (a), tetracycline (b), doxycycline (c), and minocycline (d).

The mutations that occurred more than once in independently selected mutants were most likely responsible for the tigecycline nonsusceptibility phenotype that we observed. Thus, we constructed mutants with those single mutations in the *tet*(M) sequence. All of the reconstructed mutants demonstrated some degree of reduced susceptibility to tigecycline, with MIC values ranging from 0.125 to 0.5 mg/liter at the highest *tet*(M) expression conditions (Fig. 6a). As expected, the majority of the single mutants had lower tigecycline MICs than the mutants carrying additional changes in the *tet*(M) sequence. However, a single deletion of L505 caused the highest increase of tigecycline MIC (0.5 mg/liter), even higher than strains with two amino acid substitutions. Additionally, we observed a strong functional trade-off in this mutant versus earlier classes of tetracyclines (Fig. 6b to d). Thus, the L505del mutant showed almost no activity toward tetracycline and doxycycline, while it weakly increased the MIC of minocycline. Other single amino acid substitution mutants behaved slightly differently: MICs of the single mutants S310P and Q620R were at the level of the unmutagenized control or slightly higher (Fig. 6b to d), while the S508G/R single-mutant MICs of tetracycline and minocycline were lower than that of unmutagenized Tet(M) (Fig. 6b and d). The S508G mutant tolerated higher concentrations of doxycycline than the S508R mutant (Fig. 6c), whereas the double mutants carrying S508G/R and S310P mutations were similar to the unmutagenized control with regard to tetracycline and doxycycline resistance but showed a slight reduction in resistance to minocycline. The combination of S508R and Q620R generated the lowest MICs of earlier classes of tetracyclines out of all of the double mutants tested. These results show that the Tet(M) mutants selected for increased tigecycline resistance often lose activity against earlier tetracycline antibiotics and that the mutations causing the highest increase in the tigecycline MIC also are associated with the most significant reductions in resistance against other tetracyclines.

### Potential of the Tet(X) flavin-dependent monooxygenase to develop tigecycline resistance.

**FIG 7:**
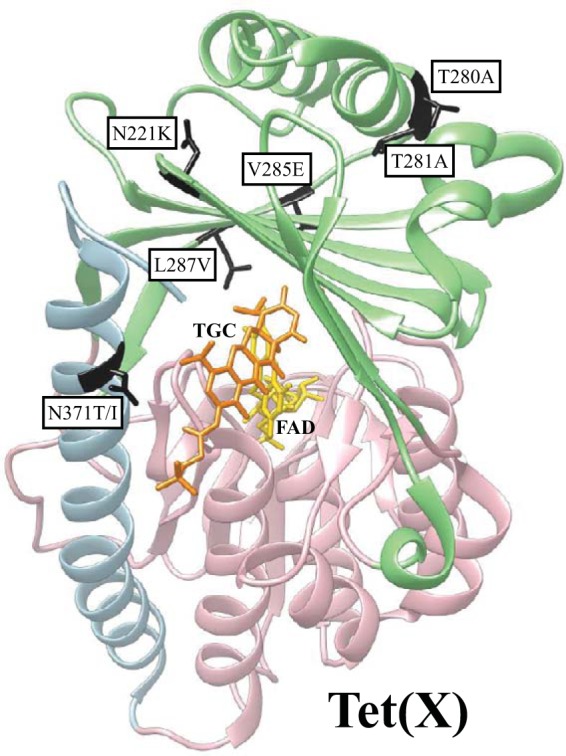
Location of tigecycline (TGC) resistance mutations in Tet(X): FAD binding domain (pink), substrate binding domain (light green), and C-terminal helix (light blue) are indicated. Mutations mentioned in the text are marked in black. TGC (orange) and FAD (gold) are indicated. The crystal structure of Tet(X) (PDB accession number 4A6N) is based on data from reference 41.

The unmutagenized version of Tet(X) had a low activity against tigecycline (Fig. 1a), and, to examine the capacity of this enzyme to develop higher-level tigecycline resistance, approximately 2.2 × 10^7^ transformants were screened on agar plates with increasing concentrations of tigecycline from two independent mutagenic libraries (TETX4L1 and TETX6L1) (see Table S2 in the supplemental material). In total, 15 unique Tet(X) mutants with increased resistance levels to tigecycline were selected, and more than half of them contained the amino acid substitution T280A as a single mutation or in combination with other changes (Fig. 7; see also Table S3 in the supplemental material). Other mutations that were identified in independent mutants more than once were N371T/I and N221K. The selected mutations were found in both domains and the C-terminal α helix of the Tet(X) enzyme, with a small cluster in the region of helix α9 and strand β16.

**FIG 8:**
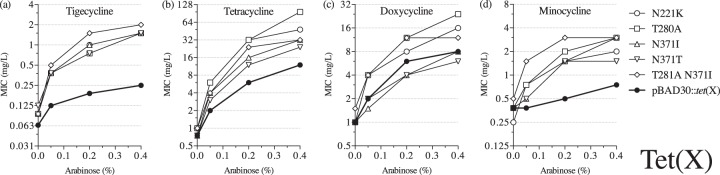
MICs of tetracyclines in E. coli expressing mutant Tet(X) proteins: tigecycline (a), tetracycline (b), doxycycline (c), and minocycline (d).

We reconstructed the four most commonly appearing mutations (T280A, N221K, N371T, and N371I) in Tet(X) and measured the MIC of tigecycline at different levels of expression for the four single reconstructed mutants and for a representative double mutant (T281A N371I) (Fig. 8). All mutants behaved similarly and, with higher expression levels, they demonstrated significantly increased MICs of tigecycline compared with the unmutagenized control (Fig. 8a). The double mutant (T281A N371I) showed the highest resistance, with MICs of tigecycline as high as 2 mg/liter. In contrast to the other *tet* genes, Tet(X) mutants largely retained, and sometimes even increased, the activity against earlier classes of tetracyclines (Fig. 8b to d). All tested mutants had MICs higher than the unmutagenized enzyme for both tetracycline and minocycline (Fig. 8b and d). However, in this case, the double mutant did not demonstrate the highest increase of the tetracycline MIC, and mutants with alterations in position N371 showed the lowest tetracycline MICs, which were still higher than the parental Tet(X). Similarly, mutations in position N371 negatively affected the activity of Tet(X) against doxycycline (Fig. 8c). Overall, the activity of Tet(X) can be substantially improved to effectively inactivate tigecycline, and, in most cases, this does not reduce the ability of the enzyme to modify earlier-class tetracylines.

## DISCUSSION

We investigated the potential of the most common tetracycline antibiotic resistance mechanisms encoded by *tet* genes to develop resistance to tigecycline. None of the unmutagenized Tet proteins showed any clinically relevant increase in tigecycline MICs when expressed from a P_BAD_ promoter, even though weak activity was observed with the Tet(A) and Tet(X) proteins. To further explore the potential of these proteins to confer tigecycline resistance, we generated mutagenized sequence libraries for all four *tet* determinants and screened for mutants with increased tigecycline MICs. Results showed that increased tigecycline MICs could be achieved with all of the Tet proteins, although the level of increase varied extensively.

Tet(A) is an efflux protein that is anchored in the inner membrane by 12 putative transmembrane α helices, which are connected by cytoplasmic and periplasmic loops (Fig. 2). The starting *tet*(A) sequence that we used as a template for mutagenesis in this study had a double frameshift mutation leading to amino acid substitutions in the interdomain loop C3 that have been suggested to cause an increase in tigecycline resistance in Salmonella isolates (19, 24), but a recent study indicated conflicting results (34). In our study, a majority of the amino acid substitutions leading to reduced tigecycline susceptibility accumulated in the predicted transmembrane regions TM2, TM4, TM5, TM7, TM8, TM10, and TM11 that are suggested to line the channel in the related Tet(B) pump (35). No mutations were found in the putative transmembrane regions TM1, TM3, TM6, TM9, and TM12, predicted to have a structural function in the Tet(B) transporter (35). Interestingly, we observed substitutions in two conserved glycines (G152 and G249), which are essential for Tet(B) function (35). G152 was earlier shown to be important for pumping out tetracycline by Tet(C) (36). Tet(A) amino acid M307, A342, and I349 homologues were shown to be of moderate importance to the Tet(B) structure and/or function (35). In our study, the mutations at these positions contributed to the reduced susceptibility of tigecycline. Two other Tet(A) amino acid substitutions were involved in elevated tigecycline MICs (R299Q and G300E). Both of these amino acids are conserved in Tet(C), and mutations R299Q and G300D were previously selected as suppressors for G71D/S and A322T changes leading to reduced efflux of tetracycline (36, 37).

Collectively, our results are in agreement with others (24, 38) showing the importance of changes in the efflux channel architecture to accommodate the bulky side chain of glycylcyclines at position C-9. It is also evident that these changes are often associated with a functional trade-off and, as exemplified in this study and earlier reports (24, 38), that such mutants show impaired transport of the original substrates.

In order to determine if Gram-positive efflux pumps also can transport tigecycline, we screened for Tet(K) mutants with reduced susceptibility to tigecycline. We were able to select a few mutants with modest increases of tigecycline MIC values. One mutation that was identified in two independent mutants was Y58H. It is located in the putative TM2 region, which is one of the predicted channel-forming segments. Furthermore, this tyrosine is a conserved amino acid in Tet(A), Tet(B), and Tet(C) pumps, and a similar amino acid, phenylalanine, is present in the corresponding position of Tet(L). Another amino acid substitution, S73F, was present next to D74, which has been shown to play an important role in tetracycline transport by Tet(K) (39). Overall, it was comparably harder to select Tet(K) than Tet(A) mutants. The few Tet(K) mutants that demonstrated reduced susceptibility to tigecycline were also poorer at exporting the original substrates, similar to what we observed for Tet(A) mutants.

It has been reported that tigecycline inhibits protein synthesis even if the ribosomal protection protein Tet(M) is present (2). Loop III of domain IV has been suggested to play a key role in displacing tetracyclines from their binding site in the 30S subunit of the ribosome (6, 10, 11). To our knowledge, no tigecycline-resistant Tet(M) proteins have been previously reported, but we identified here a set of Tet(M) mutants with reduced susceptibility to tigecycline. We selected Tet(M) mutants carrying mutations in two positions of loop III, L505 and S508 (Fig. 5). The highest tigecycline MIC values were observed in mutants carrying a deletion of L505, which results in a shortened loop III in the Tet(M) protein. At the same time, this mutation abolished Tet(M) activity on tetracycline in accordance with shortening of the corresponding loop in Tet(O) that has been shown to cause the inability of the protein to protect the ribosome from tetracycline (40). Another loop III position that we frequently found changed to either glycine or arginine in the selected mutants was S508. It has been demonstrated that S508 is a conserved serine (11); nevertheless, single S508A and triple S508A P509A V510A mutants can produce active Tet(M) proteins, as shown earlier (10). These authors suggested that the changes within loop III are acceptable as long as the loop conformation is maintained (11). In addition to changes in domain IV, we identified two more mutations that were isolated more than once in independent mutants and contributed to the increased resistance level to tigecycline. Q620R was located in the CTE, the part of Tet(M) that contains the C-terminal helix, which is important for Tet(M) function (10). The second amino acid substitution, S310P, was present in domain II, which directly interacts with 16S rRNA (11). These changes, in combination with substitutions S508G/R, caused elevated tigecycline MICs. Overall, our results show that it is possible to select ribosomal protection proteins with reduced susceptibility to tigecycline, but, in many cases, these changes come with collateral sensitivity to earlier tetracyclines, as observed for the efflux systems discussed above.

Last, we examined the Tet(X) enzymatic modification resistance mechanism and its ability to provide resistance to tigecycline. This enzyme is active against tigecycline, with the MIC reported to be 2 mg/liter when expressed on the high-copy-number vector pUC19 (14). We selected Tet(X) mutants with elevated MICs of tigecycline, and, once overexpressed, some of the mutant Tet(X) enzymes provided clinically relevant resistance. In most cases, these mutants did not show any functional trade-off with regard to activity toward earlier-class tetracyclines, and their activities were improved toward all tested tetracyclines. The most common amino acid substitutions isolated in our study were T280A, N371T/I, and N221K (Fig. 7). All of them were present in the substrate-binding domain (D2), but only N371T/I mutations were facing the active site. The other two amino acid residues were closer to the putative O_2_ binding pockets. T280A has been previously suggested to interfere with O_2_ diffusion (41). Therefore, other mutations (T281A, V285E, and L287V) from our screen that are located close to those hydrophobic pockets might also affect O_2_ transport within the enzyme. Furthermore, T280A and N371T/I changes were also selected in an earlier study, in which minocycline was used as a selective agent (42). In agreement with that study, we also observed that these mutations conferred higher resistance to minocycline. These results show that Tet(X) has the potential to evolve high-level resistance to tigecycline without compromising its activity against early tetracyclines.

In conclusion, all Tet proteins that were tested can mutate to acquire high-level [Tet(A) and Tet(X)] or low-level [Tet(K) and Tet(M)] resistance to tigecycline. Importantly, for the Tet(A), Tet(K), and Tet(M) proteins, an increased capacity to resist tigecycline is associated with a collateral loss of some or all of the initial activity against the first two classes of tetracyclines. This could be a useful feature to exploit from a therapeutic standpoint, suggesting that earlier-class tetracyclines could be used to treat tigecycline-resistant Tet-mutant bacterial infections. However, troublesome trends are observed for the Tet(X) enzyme. Thus, if one compares these different resistance mechanisms and their propensities to develop clinically relevant resistance, one would predict that the Tet(X) mechanism would potentially be the most problematic in the future with regard to tigecycline resistance. Since the Tet(X) enzyme has a weak intrinsic activity that can be improved by at least four different amino acid substitutions, clinically relevant resistance levels (as set by the European Committee on Antimicrobial Susceptibility Testing) can be reached for all types of tetracyclines without collateral loss in activity. Furthermore, as Tet(X) can spread through horizontal gene transfer (15), this tetracycline resistance mechanism in combination with currently identified chromosomal tigecycline resistance mechanisms could potentially compromise the whole class of tetracycline drugs in the future.

## Supplementary Material

Supplemental material
