# Supplementary material for: Potential of Tetracycline Resistance Proteins To Evolve Tigecycline Resistance
Source: Antimicrob Agents Chemother. 2016 Jan 29;60(2):789–96. doi: 10.1128/AAC.02465-15 (PMC4750697; doi:10.1128/AAC.02465-15)
Supplement: Supplemental material [file supp_60_2_789__index.html]

Potential of Tetracycline Resistance Proteins To Evolve Tigecycline Resistance — Supplemental material 

# Potential of Tetracycline Resistance Proteins To Evolve Tigecycline Resistance

## Supplemental material

- Supplemental file 1 -

  Supplemental Tables S1 to S3

  PDF, 131K
