# Supplementary material for: Potential of Tetracycline Resistance Proteins To Evolve Tigecycline Resistance
Source: Antimicrob Agents Chemother. 2016 Jan 29;60(2):789–96. doi: 10.1128/AAC.02465-15 (PMC4750697; doi:10.1128/AAC.02465-15)
Supplement: Supplemental material [file AAC.02465-15_zac002164794so1.pdf]

Supplementary material

Tables

TABLE S1 Primer sequences

Primer	Sequence (5’→3’)	Purpose
pBAD30_scr_F	ATAATCACGGCAGAAAAGTCCACA	Screening
pBAD30_scr_R	GGAGACCCCACACTACCATC	Screening
pBAD30-F	TTAGCGGATCCTACCTGACG	Sequencing
pBAD30-R	ACGGCGTTTCACTTCTGAGT	Sequencing
tetM_seq	AAACCAATGGAAGCCCAGAAA	Sequencing
tetX_seq	CTGCTGTTTCACTCGGTTTATT	Sequencing
tetA_pUUH_new_F	CAGCAGAATTCGTAACAGAGAAAGAGGAGAGCAGACGTGAAACCCAACAGA	Cloning
tetA_pUUH_R	GTATCTAGAAGGTTGATCAGCGATCGGCTCGTT	Cloning
tetK_clon_F	GATGAATTCCGACTAGAGAAAGAGGAGATAAAAATTGTTTAGTTTATATAAAAAATTTAAAG	Cloning
tetK_clon_R	ATATCTAGAAGGTTAACTATTCAAACTGCTTTTCAGAAC	Cloning
tetM_clon_F	GATGAATTCCGACTAGAGAAAGAGGAGATACCACATGAAAATTATTAATATTGGAG	Cloning

tetM_clon_R	ATATCTAGAACGTTACTAAGTTATTTTATTGAACATATATCTTACTT	Cloning
tetX_clon_F	AGCGAATTCAGAGAAAGAGGAGAAATACTAGATGACAATGCGAATAG	Cloning
tetX_clon_R	CAGGTCGACTCTAGATTATTATACATTTAACAATT	Cloning
tetM_S310P_F	**C**CTGGAGAAATTGTTATTTTGCA	Reconstruction
tetM_S310P_R	ATAAGCTCTATCAATCTTACATAATTCACC	Reconstruction
tetM_L505_F3	ACTATAGCCCTGTTAGTACTC	Reconstruction
tetM_L505_R3	AACCGTACTTAAAACAGATT	Reconstruction
tetM_S508G-R_F3	GCCCTGTTAGTACTCCAGC	Reconstruction
tetM_S508G_R3	**C**ATAGTATAAACCGTACTTAAAACAGAT	Reconstruction
tetM_S508R_R3	**G**ATAGTATAAACCGTACTTAAAACAGAT	Reconstruction
tetM_Q620R_F	**G**GACCCGTCGTCTAAATAGTCG	Reconstruction
tetM_Q620R_R	GGCAAACAGGTTCGCCA	Reconstruction
tetX_N221K_F	**A**TTATTATTTGCTAACCCCAATAATAATG	Reconstruction
tetX_N221K_R	TTACCTTGGTGAGATGCCATTAG	Reconstruction
tetX_T280A_F	**G**CGACGTTGTCATTTGTAGGA	Reconstruction
tetX_T280A_R	ATGAATCAATTCTTTGTAGCGTTC	Reconstruction
tetX_N371I_F	**T**CGAAATTGAAATGTTTAAACCC	Reconstruction
tetX_N371T_F	**C**CGAAATTGAAATGTTTAAACCC	Reconstruction

tetX_N371I-T_R	TTTGAGTTGATTCTTCTTGTGCT	Reconstruction

Nucleotides introducing mutation in a *tet* gene marked in bold.

TABLE S2 Characteristics of mutagenized sequence libraries

	tet	Insert	Selection	Mutagenesis	Transformants ($x10^6$ CFU)		Mutants	
Library	insert	size (bp)	step	level (SNP/kb)	Total	Tet^r	Total	Unique
TETA4L2	*tet*(A)	1200	1	0.6	7.6	5.4	16	10[a]
TETA6L1	*tet*(A)	1200	1	1.2	8.2	5.8	37	25[a]
TETK4L2	*tet*(K)	1380	1	2.5	1.1	0.26	3	3
TETK6L2	*tet*(K)	1380	1	3.3	1.6	0.28	0	0
TETM4L2	*tet*(M)	1920	1	1.6	10	1.2	6	5
TETM6L2	*tet*(M)	1920	1	2.7	10	1	9	8
TETX4L1	*tet*(X)	1167	1	2.0	12	5.7	9	9
TETX6L1	*tet*(X)	1167	1	2.6	9.5	4.2	7	6

Tet^r, tetracycline resistant.

[a]The same mutants found from several different libraries.

TABLE S3 Characteristics of Tet mutants

Strain number	Tet determinant	Vector	Library	Nucleotide change	Amino acid change	Location[a]	TGC MIC (mg/L)[b]
DA44405	Tet(A)	pBAD30	TETA4L2	T344C	V115A	TM4	1.5
DA44406	Tet(A)	pBAD30	TETA4L2	G899A	**G300E**[c]	P5	1
DA44407	Tet(A)	pBAD30	TETA4L2	A703T	**I235F**[c]	TM7	1.5
DA44408	Tet(A)	pBAD30	TETA4L2	A742C	**I248L**	TM8	1
DA44409	Tet(A)	pBAD30	TETA4L2	A460G	**M154V**	TM5	1
				T717C	-	-	
DA44410	Tet(A)	pBAD30	TETA4L2	C804T	-	-	1
				G899A	**G300E**	P5	
DA44411	Tet(A)	pBAD30	TETA4L2	G899A	**G300E**	P5	1
				A1030G	T344A	TM11	
DA44412	Tet(A)	pBAD30	TETA4L2	T183C	-	-	1.5
				G899A	**G300E**	P5	
DA44413	Tet(A)	pBAD30	TETA4L2	A460G	**M154V**	TM5	1
				G691A	A231T	TM7	
				G709A	G237S	TM7	

DA44414	Tet(A)	pBAD30	TETA4L2	G606A	-	-	1.5
				A736T	T246S	TM8	
				G899A	**G300E**	P5	
DA44415	Tet(A)	pBAD30	TETA6L1	T751G	**S251A**[c]	TM8	1.5
DA44416	Tet(A)	pBAD30	TETA6L1	G899A	**G300E**[c]	P5	1
DA44417	Tet(A)	pBAD30	TETA6L1	A460G	**M154V**	TM5	1
DA44418	Tet(A)	pBAD30	TETA6L1	G455C	G152A	TM5	1
DA44419	Tet(A)	pBAD30	TETA6L1	A703T	**I235F**[c]	TM7	1
DA44420	Tet(A)	pBAD30	TETA6L1	T704C	I235T	TM7	1.5
DA44421	Tet(A)	pBAD30	TETA6L1	T336G	-	-	1
				T751G	**S251A**	TM8	
DA44422	Tet(A)	pBAD30	TETA6L1	T164C	M55T	TM2	1
				G899A	**G300E**	P5	
DA44423	Tet(A)	pBAD30	TETA6L1	C754A	L252I	TM8	1.5
				G899A	**G300E**	P5	
DA44424	Tet(A)	pBAD30	TETA6L1	T750G	**S251A**	TM8	1
				G1008T	-	-	

DA44425	Tet(A)	pBAD30	TETA6L1	G606A	-	-	1
				T920C	**M307T**	TM10	
DA44426	Tet(A)	pBAD30	TETA6L1	A769G	I257V	TM8	1
				G899A	**G300E**	P5	
DA44427	Tet(A)	pBAD30	TETA6L1	C650G	A217G	TM7	1.5
				G899A	**G300E**	P5	
DA44428	Tet(A)	pBAD30	TETA6L1	A703T	**I235F**	TM7	1.5
				A1017T	-	-	
DA44429	Tet(A)	pBAD30	TETA6L1	C870T	-	-	1.5
				G899A	**G300E**	P5	
DA44430	Tet(A)	pBAD30	TETA6L1	G899A	**G300E**	P5	1.5
				T970C	-	-	
DA44431	Tet(A)	pBAD30	TETA6L1	A788T	Q263L	TM8	1
				A1030T	T344S	TM11	
DA44432	Tet(A)	pBAD30	TETA6L1	C231T	-	-	1.5
				T282C	-	-	
				G899A	**G300E**	P5	

DA44433	Tet(A)	pBAD30	TETA6L1	C561T	-	-	1
				T751G	**S251A**	TM8	
				G896A	R299Q	P5	
DA44434	Tet(A)	pBAD30	TETA6L1	C577T	R193C	C3	1
				G899A	**G300E**	P5	
				C1140T	-	-	
DA44435	Tet(A)	pBAD30	TETA6L1	G382A	E128K	C2	1.5
				A441G	-	-	
				G899A	**G300E**	P5	
DA44436	Tet(A)	pBAD30	TETA6L1	C885T	-	-	1
				G899A	**G300E**	P5	
				T920C	**M307T**	TM10	
DA44437	Tet(A)	pBAD30	TETA6L1	T749C	I250T	TM8	1.5
				C791T	A264V	TM8	
				C1070A	A357E	TM11	
DA44438	Tet(A)	pBAD30	TETA6L1	G745A	G249S	TM8	1.5
				G753A	-	-	

				C1025T	A342V	TM11	
DA44439	Tet(A)	pBAD30	TETA6L1	C735A	-	-	1
				G899A	**G300E**	P5	
				A1045G	I349V	TM11	
				A1087G	I363V	P6	
				T1125C	-	-	
DA44443	Tet(K)	pBAD30	TETK4L2	T172C	**Y58H**	TM2	0.19
				G177A	M59I	TM2	
DA44444	Tet(K)	pBAD30	TETK4L2	T172C	**Y58H**	TM2	0.19
				C218T	S73F	C1	
				T602C	I201T	TM7	
				T795A	-	-	
				T1134A	-	-	
DA44445	Tet(K)	pBAD30	TETK4L2	T67A	S23T	TM1	0.19
				T377C	V126A	TM4 C2	
				T1038C	-	-	
DA44446	Tet(M)	pUCBAD	TETM4L2	A1255G	I419V	IV	0.094

				A1494G	-	-	
				C1524A	**S508R**	IV L3	
DA44447	Tet(M)	pUCBAD	TETM4L2	T405A	-	-	0.094
				T928C	**S310P**	II	
				T1262A	M421K	IV	
				A1412G	Q471R	IV	
				A1522G	**S508G**	IV L3	
DA44448	Tet(M)	pUCBAD	TETM4L2	A783G	-	-	0.38
				T1470C	-	-	
				TAT1514_1516del	**L505del**	IV L3	
				A1837T	T613S	CTE	
DA44449	Tet(M)	pUCBAD	TETM4L2	G806A	R269H	II	0.125
				A1076G	E359G	III	
				A1522C	**S508R**	IV L3	
DA44450	Tet(M)	pUCBAD	TETM4L2	A778G	K260E	II	0.125
				A1522G	**S508G**	IV L3	
DA44451	Tet(M)	pUCBAD	TETM6L2	T493A	C165S	I	0.25

				A1004G	Q335R	II	
				TAT1514_1516del	**L505del**	IV L3	
DA44452	Tet(M)	pUCBAD	TETM6L2	A862G	K288E	II	0.125
				C1030A	P344T	III	
				C1524G	**S508R**	IV L3	
				A1539G	-	-	
DA44453	Tet(M)	pUCBAD	TETM6L2	T236A	M79K	I	0.125
				T412C	S138P	I	
				G589A	A197T	I	
				A1052T	E351V	III	
				C1064T	P355L	III	
				T1167G	-	-	
				T1500C	-	-	
				A1522C	**S508R**	IV L3	
				A1589G	K530R	IV	
DA44454	Tet(M)	pUCBAD	TETM6L2	A217T	I73L	I	0.125
				T330C	-	-	

				T409C	-	-	
				A1028T	H343L	III	
				A1443G	I481M	IV	
				C1524A	**S508R**	IV L3	
DA44455	Tet(M)	pUCBAD	TETM6L2	A414G	-	-	0.19
				A1522C	**S508R**	IV L3	
				T1784C	F595S	V	
DA44456	Tet(M)	pUCBAD	TETM6L2	A58T	T20S	I	0.125
				T928C	**S310P**	II	
				C1524A	**S508R**	IV L3	
DA44457	Tet(M)	pUCBAD	TETM6L2	C1524A	**S508R**	IV L3	0.19
				A1698G	-	-	
				A1859G	**Q620R**	CTE	
DA44458	Tet(M)	pUCBAD	TETM6L2	A156G	-	-	0.19
				T975C	-	-	
				A1522C	**S508R**	IV L3	
				A1656G	-	-	

				A1752G	-	-	
				A1859G	**Q620R**	CTE	
DA38587[d]	Tet(M)	pUCBAD	-	TAT1514_1516del	**L505del**	IV L3	0.38
DA38583[d]	Tet(M)	pUCBAD	-	A1522G	**S508G**	IV L3	0.125
DA38586[d]	Tet(M)	pUCBAD	-	A1522C	**S508R**	IV L3	0.125
DA40424[d]	Tet(M)	pUCBAD	-	T928C	**S310P**	II	0.064
DA40425[d]	Tet(M)	pUCBAD	-	A1859G	**Q620R**	CTE	0.094
DA40426[d]	Tet(M)	pUCBAD	-	T928C	**S310P**	II	0.094
				A1522G	**S508G**	IV L3	
DA40427[d]	Tet(M)	pUCBAD	-	T928C	**S310P**	II	0.19
				A1522C	**S508R**	IV L3	
DA40428[d]	Tet(M)	pUCBAD	-	A1522C	**S508R**	IV L3	0.25
				A1859G	**Q620R**	CTE	
DA44459	Tet(X)	pBAD30	TETX4L1	A838G	**T280A**	D2 α9	0.75
				T854A	V285E	D2 β16	
DA44460	Tet(X)	pBAD30	TETX4L1	T171C	-	-	0.5
				A838G	**T280A**	D2 α9	

				T879C	-	-	
				T1005C	-	-	
DA44461	Tet(X)	pBAD30	TETX4L1	A838G	**T280A**	D2 α9	0.75
DA44462	Tet(X)	pBAD30	TETX4L1	G55A	V19I	D1 β1	0.75
				A162G	-	-	
DA44463	Tet(X)	pBAD30	TETX4L1	C5T	T2I	D1	1
				T276C	-	-	
				A522G	-	-	
				T663G	**N221K**	D2 β14	
DA44464	Tet(X)	pBAD30	TETX4L1	T663A	**N221K**	D2 β14	3
				T859G	L287V	D2 β16	
				A1068G	-	-	
				A1112C	**N371T**	α12	
DA44465	Tet(X)	pBAD30	TETX4L1	T982A	L328M	D1 α11	3
				A1112C	**N371T**	α12	
DA44466	Tet(X)	pBAD30	TETX4L1	A155T	E52V	D1	3
				A239G	Y80C	D1 α3	

				A1112C	**N371T**	α12	
DA44467	Tet(X)	pBAD30	TETX4L1	A838G	**T280A**	D2 α9	3
				T954C	-	-	
DA44468	Tet(X)	pBAD30	TETX6L1	T36C	-	-	0.75
				A838G	**T280A**	D2 α9	
DA44469	Tet(X)	pBAD30	TETX6L1	A305G	K102R	D2	0.75
				A798G	-	-	
				A838G<br>G840A	**T280A**	D2 α9	
DA44470	Tet(X)	pBAD30	TETX6L1	G462A	-	-	1
				T729G	D243E	D2	
				A1051G	K351E	α12	
				A1112T	**N371I**	α12	
DA44471	Tet(X)	pBAD30	TETX6L1	A841G	T281A	D2	1.5
				A1112T	**N371I**	α12	
DA44472	Tet(X)	pBAD30	TETX6L1	A84G	-	-	1.5
				A437G	K146R	D1	

				A838G	**T280A**	D2 α9	
				G985A	V329M	D1 α11	
				G1018A	D340N	D1	
DA44473	Tet(X)	pBAD30	TETX6L1	T45C	-	-	1.5
				A838G	**T280A**	D2 α9	
DA40421[d]	Tet(X)	pBAD30	-	A838G	**T280A**	D2 α9	0.5
DA40422[d]	Tet(X)	pBAD30	-	A1112T	**N371I**	α12	0.5
DA40423[d]	Tet(X)	pBAD30	-	A1112C	**N371T**	α12	0.5
DA40420[d]	Tet(X)	pBAD30	-	T663A	**N221K**	D2 β14	0.5

TGC, tigecycline; TM, transmembrane; P, periplasmic; C, cytoplasmic; I-V, domains I-V; L, loop; CTE, C-terminal extension; D1, FAD-binding domain; D2, substrate-binding domain; α, α-helix; β, β-strand; del, deletion.

[a]Tet(A) pump division into periplasmic and cytoplasmic loops and transmembrane regions is only predictive.

[b]The following L-arabinose concentrations were used for MIC measurements during selections: 0.1% for Tet(A), Tet(K) and Tet(X); 0.2% for Tet(M).

[c]The same mutants found from several different libraries.

[d]Reconstructed mutants.

Amino acid changes that are repeatedly isolated more than once in the independent mutants are in bold.
